# Association between performance measures and clinical outcomes in patients with heart failure in China: Results from the HERO study

**DOI:** 10.1002/clc.24233

**Published:** 2024-02-20

**Authors:** Shiyue Zheng, Li Li, Chao Jiang, Liu He, Yiwei Lai, Wenjie Li, Xiaoyan Zhao, Xiaofang Wang, Ling Li, Xin Du, Changsheng Ma, Jianzeng Dong

**Affiliations:** ^1^ Department of Cardiology, Beijing AnZhen Hospital Capital Medical University Beijing China; ^2^ Department of Cardiology The First Affiliated Hospital of Zhengzhou University Zhengzhou China; ^3^ Department of Cardiology, Beijing AnZhen Hospital, National Clinical Research Centre for Cardiovascular Diseases, Beijing Advanced Innovation Center for Big Data‐Based Precision Medicine for Cardiovascular Diseases Capital Medical University Beijing China

**Keywords:** heart failure, HERO study, mortality, performance measure, readmission

## Abstract

**Background:**

There is great heterogeneity in the quality of care among hospitals in China, but studies on the performance measures and prognosis of patients with heart failure (HF) are still deficient.

**Hypothesis:**

Performance measures have been used as a guideline to clinicans, however, the association between them and outcomes among HF patients in China remains unclear.

**Methods:**

We analyzed 4497 patients with HF from the Heart Failure Registry of Patient Outcomes study. Performance measures were determined according to the guidelines, and the patients were divided into four groups based on a composite performance score. Multiple imputation and Cox proportional‐hazard regression models were used to assess the association between the performance measures and clinical outcomes.

**Results:**

Overall, only 12.5% of patients met the top 25% of the performance measures, whereas 33.5% of patients met the bottom 25% of the measures. A total of 992 (22.2%) patients died within 1 year, involving a larger proportion of patients who had met only the bottom 25% of the performance measures than had met the top 25% (27.0% vs. 16.3%, respectively). The patients who met the top 25% of the measures had a lower 1‐year mortality rate (adjusted hazard ratio: 0.78, 95% confidence interval: 0.61–0.98).

**Conclusions:**

The association between performance measures and mortality appeared to follow a dose–response pattern with a larger degree of compliance with performance measures being associated with a lower mortality rate in patients with HF. Accordingly, the quality of care for patients with HF in China needs to be further improved.

**Abbreviations**
ACEI: angiotensin‐converting enzyme inhibitor
ARB: angiotensin II receptor blocker
ARNI: angiotensin receptor blockers and neprilysin inhibitor
BNP: brain natriuretic peptide
HERO: Heart Failure Registry of Patient Outcomes
HF: heart failure
LVEF: left ventricular ejection fraction
MRA: mineralocorticoid receptor antagonist
NT‐proBNP: N‐terminal prohormone of BNP

## 1 INTRODUCTION

Heart failure (HF) is the final stage of all cardiovascular diseases. The total number of patients with HF continues to grow owing to population growth and aging, with an estimated 64.3 million affected people worldwide, and the average age of onset has decreased.^1^, ^2^ HF remains a major clinical and public health problem, with extremely high mortality and readmission rates.^3^ Pharmacological therapies, including angiotensin‐converting enzyme inhibitors (ACEIs)/angiotensin II receptor blockers (ARBs)/angiotensin receptor blockers and neprilysin inhibitors (ARNIs), beta‐blockers, and mineralocorticoid receptor antagonists (MRAs), have been included in HF management guidelines based on the results of randomized controlled trials.^4^ Although the efficacy of these drugs has been demonstrated, the real‐world mortality and readmission rates owing to HF remain high because of the gap between clinical practice and guidelines.^5^, ^6^, ^7^ This has gradually increased the financial burden of patients with HF, especially in low‐and middle‐income countries.^8^, ^9^, ^10^ Patients with HF in China have the highest medical burden among those in low‐ and middle‐income countries at RMB 28 974 ($4199).^11^, ^12^

Over the past 20 years, efforts have been made to measure, report, and improve the quality of care for patients with HF to reduce the burden of mortality and readmission. In 2005, the Get With the Guidelines‐Heart Failure (GWTG‐HF) registry was launched in the United States to improve the quality of care for patients with HF and reduce heterogeneity among hospitals.^13^ In the past decade, the Chinese healthcare system has also improved the quality of care of patients with HF, and suitable quality measures for the Chinese medical system have been formulated. Although studies have revealed significant differences in performance measures among hospitals,^14^, ^15^ current evidence for the relationship between these measures and clinical outcomes is insufficient. To address this issue, we analyzed the association between performance measures and clinical outcomes of patients with HF in China.

## 2 METHODS

### 2.1 Study population

In the Heart Failure Registry of Patient Outcomes (HERO) study, a prospective, longitudinal, seasonally rotating, multicenter registry study, 5620 patients diagnosed with HF at 73 hospitals in Henan Province from November 2017 to November 2018 were recruited.^7^ These hospitals included provincial‐, municipal‐, and county‐level hospitals that cover different geographical areas and with different sizes of referral populations. Baseline data were collected by trained cardiologists to obtain information on patients’ sociodemographic characteristics, medical history, diagnosis, and treatment. We excluded patients who were lost to follow‐up and those who died in the hospital.

### 2.2 Performance measures and clinical outcomes

The five performance measures we used were based on the 2020 American College of Cardiology/American Heart Association (AHA) clinical performance and quality measures for adults with HF and the expert consensus on clinical performance and quality measures for adults with HF in China and were as follows^16^, ^17^: (1) the brain natriuretic peptide (BNP)/N‐terminal prohormone of BNP (NT‐proBNP) test; (2) left ventricular ejection fraction (LVEF) assessment; (3) beta‐blocker therapy; (4) ACEI/ARB/ARNI therapy; and (5) MRA therapy. We computed a composite score to reflect the hospital quality of care for HF, calculated as the number of performance measures actually completed by each patient divided by the number of measures that patients should theoretically complete. Based on composite score of performance measures, patients were further divided into quartiles: bottom 25% (*N* = 1.505), 25%–50% (*N* = 1320), 50%–75% (*N* = 1108), and top 25% (*N* = 564). The 30‐day and 1‐year mortality and readmission rates of patients with HF were chosen as the clinical outcomes for the present study. Mortality and readmission were defined as death from any cause and hospitalization for HF after discharge, respectively.

### 2.3 Statistical analysis

Normally distributed continuous variables were presented as means ± standard deviations and compared with one‐way analysis of variance, whereas non‐normally distributed continuous variables were displayed as medians (Q1, Q3) and compared with the nonparametric Kruskal–Wallis test. Categorical variables were expressed as percentages and compared using the *χ* ^2^ test. Kaplan–Meier curves were plotted to analyze the clinical outcomes and compared using the log‐rank test. Multivariable Cox proportional‐hazards regression was used to evaluate the association between the completion of performance measures and clinical outcomes, and the models were adjusted for potential confounding variables after multiple imputations of missing data. First, we adjusted for patient baseline characteristics including age, sex, body mass index, low educational level, low income, current smoking status, current drinking status, systolic blood pressure, diastolic blood pressure, New York Heart Association classification (NYHA class), LVEF, heart rate, medical history, estimated glomerular filtration rate (eGFR), anemia, potassium concentration, and serum sodium concentration.^18^, ^19^, ^20^, ^21^ Second, we adjusted for prescriptions for digoxin, diuretics, acetylsalicylic acid, clopidogrel, statins, and nonvitamin K antagonist oral anticoagulants (NOACs) at discharge. All data analyses were performed using IBM SPSS Statistics for Windows version 27.0 (IBM Corp.).

## 3 RESULTS

### 3.1 Baseline characteristics

**Figure 1:**
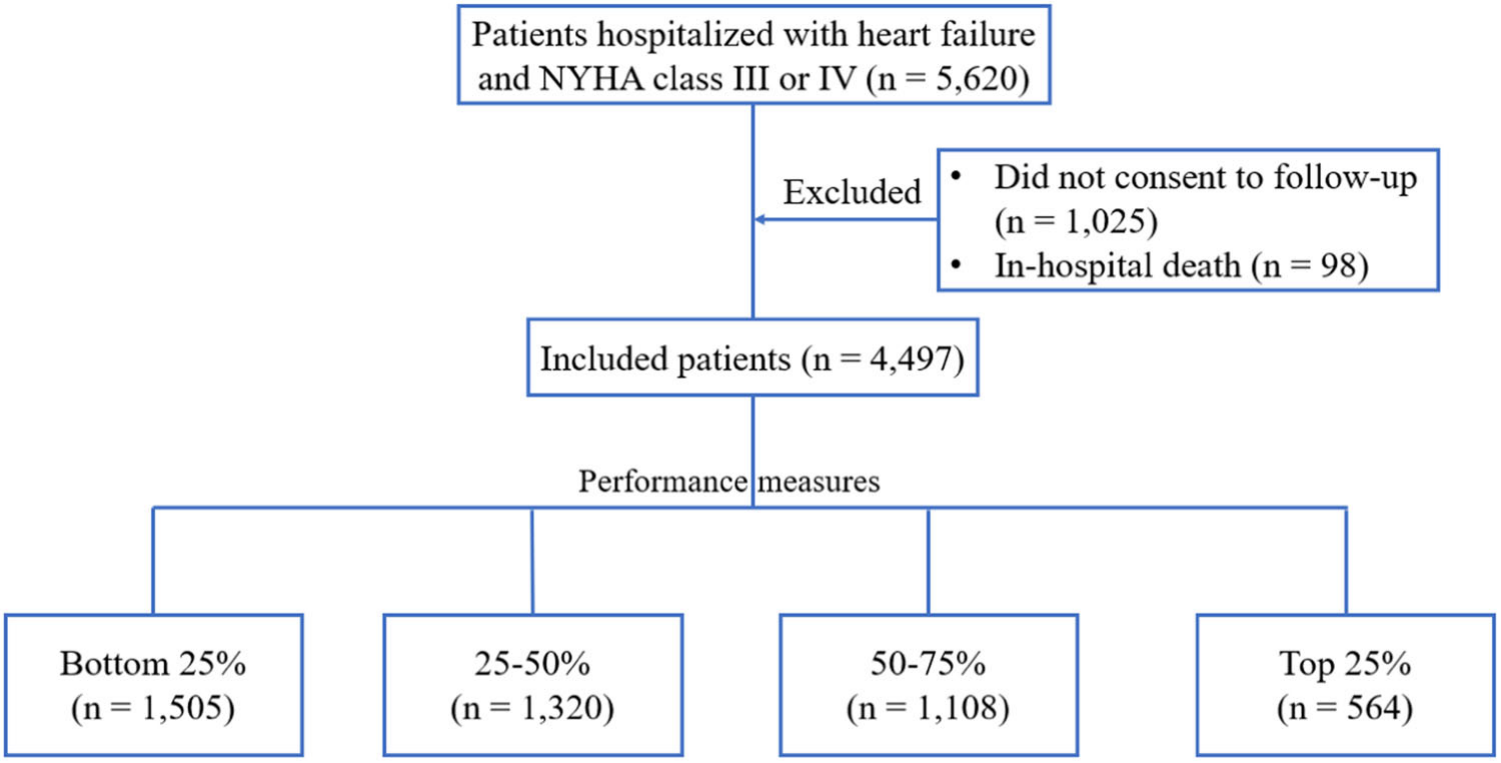
Flowchart. NYHA, New York Heart Association.

Among the 5620 patients with HF and an NYHA class III or IV in the HERO study, those who died during hospitalization (*n* = 98) or were lost to follow‐up (*n* = 1025) were excluded. Hence, 4497 patients were included in this study (Figure 1). Patients in top 25% of the composite score were more likely to be men (55.4% vs. 48.1%) and younger (median age, 67 vs. 73 years), have more comorbidities (coronary heart disease [CHD], atrial fibrillation/atrial flutter [AF/AFL], diabetes mellitus [DM], or hypertension), and have HF with reduced ejection fraction (HFrEF) (36.5% vs. 18.1%), and less likely to have a decreased eGFR (17.6% vs. 24.4%) than those in bottom 25% (Table 1). Patients in the top 25% of the composite score were more likely to be treated at hospitals with more cardiologists (median, 21 vs. 15, *p* < .001), beds (median, 124 vs. 110, *p* < .001) and the capacity for cardiac resynchronization therapy (40.4% vs. 24.0%, *p* < .001) or cardioverter‐defibrillator implantation (40.1% vs. 29.2%, *p* < .001), and more likely to be treated at tertiary hospitals (32.0% vs. 16.6%, *p* < .001) than those in the bottom 25%.

**Table 1:** Patient and hospital baseline characteristics.

Baseline characteristics	Bottom 25% (*n* = 1505)	25%–50% (*n* = 1320)	50%–75% (*n *= 1108)	Top 25% (*n *= 564)	*p* Value
Patient characteristics					
Male, *n* (%)	724 (48.1)	666 (50.5)	568 (51.3)	312 (55.4)	.028
Age (years)^a^	72.9 ± 11.6	72.5 ± 11.0	70.5 ± 12.5	67.2 ± 14.2	<.001
Age (years), *n* (%)					<.001
≥75	684 (46.2)	571 (44.1)	421 (38.7)	181 (32.4)	
65–75	465 (31.4)	436 (33.7)	383 (35.2)	174 (31.1)	
<65	330 (22.3)	287 (22.2)	285 (26.2)	204 (36.5)	
Insurance, *n* (%)					.683
Publicly funded healthcare	8 (0.5)	3 (0.2)	6 (0.5)	3 (0.5)	
UEBMI/URBMI	309 (20.8)	251 (19.3)	239 (21.9)	112 (20.2)	
NRCMS	1027 (69.2)	933 (71.8)	746 (68.3)	390 (70.3)	
Self‐finance	98 (6.6)	84 (6.5)	78 (7.1)	41 (7.4)	
Other	43 (2.9)	29 (2.2)	24 (2.2)	9 (1.6)	
Low education, *n* (%)	1033 (81.1)	906 (79.7)	738 (75.2)	359 (67.2)	<.001
Low income, *n* (%)	864 (70.5)	765 (68.9)	625 (65.3)	314 (60.6)	<.001
BMI (kg/m^2^)^a^	22.8 ± 3.6	23.0 ± 3.7	23.5 ± 3.6	23.9 ± 4.3	<.001
BMI (kg/m^2^), *n* (%)					<.001
≥28	94 (6.8)	102 (8.3)	84 (8.3)	58 (11.1)	
24–28	383 (27.9)	351 (28.7)	338 (33.4)	182 (34.7)	
<24	897 (65.3)	772 (63.0)	589 (58.3)	284 (54.2)	
Current smoking, *n* (%)	125 (8.4)	121 (9.2)	127 (11.5)	85 (15.1)	<.001
Current drinking, *n* (%)	66 (4.4)	66 (5.0)	77 (7.0)	55 (9.8)	<.001
SBP ≥ 140 mmHg, *n* (%)	534 (35.6)	510 (38.7)	509 (46.0)	234 (41.6)	<.001
DBP ≥ 90 mmHg, *n* (%)	353 (23.6)	348 (26.4)	383 (34.6)	198 (35.2)	<.001
Heart rate (bpm)^a^	87.0 ± 22.4	87.4 ± 22.5	90.4 ± 24.2	90.7 ± 21.3	<.001
LVEF, *n* (%)					<.001
≥50%	282 (63.9)	411 (58.6)	443 (52.9)	230 (40.8)	
40%–49%	79 (17.9)	131 (18.7)	183 (21.9)	128 (22.7)	
<40%	80 (18.1)	159 (22.7)	211 (25.2)	206 (36.5)	
NYHA class, *n* (%)					.301
III	803 (53.4)	696 (52.7)	551 (49.7)	295 (52.3)	
IV	702 (46.6)	624 (47.3)	557 (50.3)	269 (47.7)	
Medical history, *n* (%)					
CHD^b^	234 (15.6)	267 (20.3)	208 (18.9)	115 (20.5)	.005
AF/AFL	360 (24.0)	335 (25.4)	328 (29.8)	162 (28.7)	.004
DM	268 (17.9)	260 (19.8)	254 (23.0)	109 (19.4)	.013
Hypertension	634 (42.2)	630 (47.8)	585 (53.0)	280 (49.8)	<.001
Cerebrovascular disease^c^	211 (14.1)	187 (14.2)	181 (16.4)	92 (16.3)	.248
COPD	188 (12.5)	199 (9.0)	73 (6.6)	30 (5.3)	<.001
BNP (pg/mL)^d^	1037.1 (277.4, 4232.5)	1004.1 (415.9, 3428.9)	1057.0 (427.3, 3371.9)	942.9 (420.0, 2933.5)	.992
NT‐proBNP (pg/mL)^d^	2812.2 (637.9, 8000)	2990.0 (971.0, 8197.0)	3389.5 (1296.7, 7576.8)	3360.5 (1363.8, 7986.6)	.046
TG (mmol/L)^a^	1.3 ± 0.9	1.2 ± 0.8	1.3 ± 1.2	1.4 ± 1.1	.093
LDL‐C (mmol/L)^a^	2.3 ± 1.0	2.3 ± 0.9	2.3 ± 0.8	2.4 ± 0.8	.437
HDL‐C (mmol/L)^a^	1.2 ± 1.0	1.1 ± 0.4	1.1 ± 0.3	1.1 ± 0.3	<.001
Anemia, *n* (%)	705 (49.7)	645 (50.8)	498 (46.6)	225 (40.9)	<.001
Potassium (mmol/L)^a^	4.2 ± 0.7	4.2 ± 0.7	4.1 ± 0.6	4.1 ± 0.6	<.001
Serum sodium (mmol/L)^a^	138.5 ± 5.0	139.0 ± 4.7	139.2 ± 4.8	139.3 ± 4.4	<.001
eGFR < 60 mL/min/1.73 m^2^, *n* (%)	330 (24.4)	270 (22.3)	194 (18.7)	96 (17.6)	<.001
Hospital characteristics					
Tertiary hospital, *n* (%)	248 (16.6%)	292 (22.4%)	297 (27.1%)	179 (32.0%)	<.001
Medical university‐affiliated hospital, *n* (%)	94 (6.3%)	122 (9.3%)	137 (12.5%)	94 (16.8%)	<.001
Provincial administrative regions, *n* (%)	77 (5.2%)	91 (7.0%)	104 (9.5%)	74 (13.2%)	<.001
Cardiologists, median (Q1, Q3)	15 (10,22)	17 (11,29)	18 (11,33)	21 (12,42)	<.001
Beds in the department of cardiology, median (Q1, Q3)	110 (79, 150)	113 (80,150)	118 (90,150)	124 (81,180)	<.001
CCU, *n* (%)	1139 (76.3%)	1065 (81.6%)	921 (84.1%)	501 (89.6%)	<.001
CRT capability, *n* (%)	359 (24.0%)	400 (30.7%)	396 (36.2%)	226 (40.4%)	<.001
ICD capability, *n* (%)	436 (29.2%)	414 (31.7%)	388 (35.4%)	224 (40.1%)	<.001

Abbreviations: AF, atrial fibrillation; AFL, atrial flutter; BMI, body mass index; BNP, brain natriuretic peptide; BUN, blood urea nitrogen; CCU, coronary care unit; CHD, coronary heart disease; COPD, chronic obstructive pulmonary disease; CRT, cardiac resynchronization therapy; DBP, diastolic blood pressure; DM, diabetes mellitus; eGFR, estimated glomerular filtration rate; HDL‐c, high‐density lipoprotein cholesterol; ICD, implantable cardioverter‐defibrillator; LDL‐c, low‐density lipoprotein cholesterol; LVEF, left ventricular ejection fraction; NRCMS, new rural cooperative medical system; NT‐proBNP, N‐terminal pro‐b‐type natriuretic peptide; NYHA class, New York Heart Association classification; SBP, systolic blood pressure; TC, total cholesterol; TG, total triglycerides; UEBMI, urban employee basic medical insurance; URBMI, urban residents' basic medical insurance system.

^a^ Mean ± SD.

^b^ CHD diagnosed by coronary angiography or CTA, or previous history of myocardial infarction, or revascularization (PCI, CABG).

^c^ Including ischemic stroke, hemorrhagic stroke, and transient ischemic attack.

^d^ Median (Q1, Q3).

### 3.2 Performance measures

The measurements of LVEF and BNP/NT‐proBNP were performed in 56.5% and 80.6% of the 4497 patients, respectively (Table 2). Among 656 patients with HFrEF, the usage of ACEI/ARB/ARNI, beta‐blocker, and MRA were 52.5%, 60.5%, and 84.9% respectively, at discharge, which is much lower than in the United States except for MRA therapy.^22^

**Table 2:** Performance measures at discharge.

Performance measures, *n* (%)	All patients (*n* = 4497)	HFrEF patients (*n* = 656)	GWTG‐HF (*n *= 423 333), %
BNP/NT‐pro BNP test	3624 (80.6)	563 (85.8)	–
LVEF assessment	2543 (56.5)	656 (100)	100
Beta‐blocker therapy	2276 (51.1)	394 (60.5)	89.9
ACEI/ARB/ARNI therapy	1966 (44.3)	341 (52.5)	94.5
MRA therapy	3186 (71.5)	5553 (84.9)	41.9

Abbreviations: ACEI, angiotensin‐converting enzyme inhibitor; ARB, angiotensin II receptor blocker; ARNI, angiotensin receptor blockers and neprilysin inhibitor; BNP, brain natriuretic peptide; GWTG‐HF, Get With the Guidelines‐Heart Failure registry; HFrE, heart failure with reduced ejection fraction; LVEF, left ventricular ejection fraction; MRA, mineralocorticoid receptor antagonist; NT‐proBNP, N‐terminal prohormone of BNP.

### 3.3 Clinical outcomes

**Figure 2:**
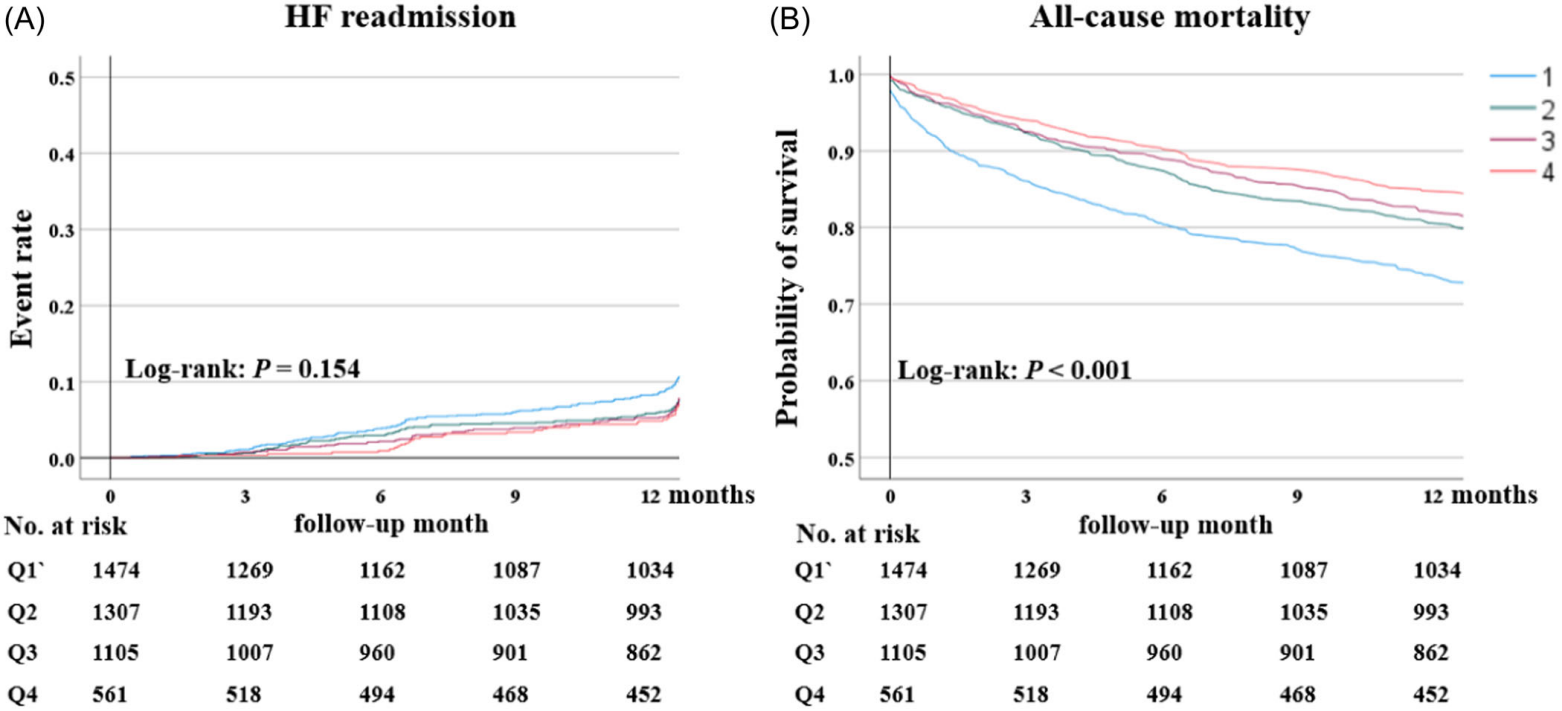
Association between performance measures and clinical outcomes. Kaplan–Meier curves for all‐cause mortality. Day 0 is the time of the first follow‐up after discharge. (A) The event rate of HF readmission; (B) the probability of freedom from all‐cause mortality. 1: Bottom 25%; 2: 25%–50%; 3: 50%–75%; 4: top 25%. HF, heart failure.

A total of 992 (22.2%) patients died and 1585 (35.2%) were readmitted for HF after a median follow‐up period of 12.9 months (interquartile range: 11.3, 13.1). Figure 2 and Table 3 show the association between performance measures and clinical outcomes. After adjusting for patient characteristics including socioeconomic factors and the use of digoxin, diuretics, acetylsalicylic acid, clopidogrel, statins, and NOACs, the risks of 30‐day and 1‐year mortality were lower by 51% (*p* = .010) and 22% (*p* = .035), respectively. For the readmission rate, the adjusted hazard ratios of patients with high‐quality care for 30 days and 1 year were 0.65 (95% confidence interval [CI]: 0.37–1.14, *p* = .131) and 0.93 (95% CI: 0.77–1.12, *p* = .436), respectively, compared with those of patients with low‐quality care.

**Table 3:** Association between performance measures and clinical outcomes after multiple imputations.

	Crude HR (95% CI)	*p* Value	Adjusted HR (95% CI)^a^	*p* Value	Adjusted HR (95% CI)^b^	*p* Value
30‐day mortality						
Q1 (reference)	1.00		1.00		1.00	
Q2	0.43 (0.31–0.60)	<.001	0.50 (0.35–0.69)	<.001	0.54 (0.39–0.76)	<.001
Q3	0.43 (0.30–0.61)	<.001	0.56 (0.39–0.80)	.002	0.65 (0.45–0.95)	.025
Q4	0.31 (0.18–0.53)	<.001	0.42 (0.25–0.73)	.002	0.49 (0.28–0.84)	.010
1‐year mortality						
Q1 (reference)	1.00		1.00		1.00	
Q2	0.72 (0.61–0.83)	<.001	0.77(0.66–0.90)	.001	0.82 (0.70–0.96)	.016
Q3	0.63 (0.53–0.74)	<.001	0.75 (0.63–0.89)	.001	0.82 (0.69–0.98)	.031
Q4	0.55 (0.44–0.69)	<.001	0.70 (0.56–0.88)	.003	0.78 (0.61–0.98)	.035
30‐day readmission						
Q1 (reference)	1.00		1.00		1.00	
Q2	0.62 (0.41–0.94)	.024	0.60 (0.40–0.91)	.016	0.60 (0.39–0.91)	.017
Q3	0.54 (0.35–0.85)	.008	0.52 (0.33–0.83)	.006	0.53 (0.33–0.85)	.008
Q4	0.69 (0.40–1.17)	.169	0.64 (0.37–1.11)	.109	0.65 (0.37–1.14)	.131
1‐year readmission						
Q1 (reference)	1.00		1.00		1.00	
Q2	0.93 (0.81–1.06)	.260	0.93 (0.81–1.06)	.250	0.92 (0.80–1.05)	.208
Q3	0.86 (0.74–0.99)	.031	0.88 (0.76–1.02)	.078	0.89 (0.77–1.03)	.114
Q4	0.89 (0.75–1.06)	.192	0.92 (0.77–1.10)	.364	0.93 (0.77–1.12)	.436

Abbreviations: BMI, body mass index; CI, confidence interval; DBP, diastolic blood pressure; eGFR, estimated glomerular filtration rate; HR, hazard ratio; LVEF, left ventricular ejection fraction; NYHA class, New York Heart Association classification; SBP, systolic blood pressure.

^a^ Adjusted for age, sex, BMI, low educational level, low income, current smoker, current drinking, SBP ≥ 140 mmHg, DBP ≥ 90 mmHg, NYHA class, LVEF, heart rate ≥ 100 bpm, medical history, eGFR < 60 mL/min/1.73 m^2^, anemia, potassium, serum sodium.

^b^ Adjusted for variables above and prescription of digoxin, diuretics, acetylsalicylic acid, clopidogrel, statins, and NOAC at discharge.

## 4 DISCUSSION

The main results in this study were as follows. First, for every patient who received high‐quality care, more than two received low‐quality care. Second, patients admitted to hospitals with more cardiologists and beds or affiliated with medical universities were more likely to receive high‐quality care. Third, the 30‐day and 1‐year mortality rates of patients receiving high‐quality care were significantly lower than those of patients receiving low‐quality care, although the readmission rates did not differ between them. This association persisted even after adjusting for relevant confounding factors.

Previous studies that have addressed the associations between process performance measures and clinical outcomes have primarily focused on patients with chronic HF and mortality in different geographical and socioeconomic areas of the world. This study from China aims to add to the current heterogeneity of healthcare for cardiovascular disease, worldwide. The HERO study, which included patients with HF in Henan Province, is one of the largest registered HF‐research cohorts in China. The data released from the seventh national census by the National Bureau of Statistics in 2021 confirmed that Henan Province is one of the most populous provinces in China, with a population of almost 100 million, accounting for 7.04% of the total population, and it is usually regarded as representative of the Chinese population.^23^ Our results demonstrated that the gap in the quality of care for HF remains large, with more than double the number of patients who receive low‐quality of care compared with those who receive high‐quality care. The LVEF was assessed in only 56.5% of patients with HF, a far cry from the 97% in Europe,^16^, ^24^ whereas the prescriptions for ACEIs/ARBs/ARNIs, beta‐blockers, and MRAs at discharge for HFrEF were 52.5%, 60.5%, and 84.9%, respectively. Despite significantly increased adherence to HF guidelines in China, it is still lower than in the United States and Europe, except for MRA therapy,^25^, ^26^, ^27^, ^28^ a conclusion that was also confirmed in the China‐HF registry study.^29^

We speculated on the main reasons for the above‐mentioned results. First, the degree of compliance with performance measures is related to patient characteristics, such as economic and educational levels: patients with a low income and educational level and more complications (CHD, AF/AFL, DM, hypertension, COPD) meet relatively few of the performance measures included in our composite score. This is in line with previous studies in which clinical compliance with the guidelines was affected by the individual's awareness of the disease and their economic level; moreover, in clinical practice, medication is limited by certain physiological factors, including heart rate, blood pressure, renal function, or complications.^30^, ^31^, ^32^, ^33^ Second, the low number of prescriptions for guideline‐recommended medications may also be attributed to the fact that LVEF is not evaluated in some patients, resulting in patients with HFrEF being overlooked. Hence, although a substantial opportunity for improvement in the performance measures themselves remains, more patients should be detected with current measures for patients with HFrEF, and heterogeneity in HF care among hospitals should be reduced. A similar conclusion was made by Gupta et al.^14^ In addition, the low utilization rate of ARNIs during the study period can probably be attributed to their relatively recent approval for the market in China (2017): they were not included in the national health insurance catalog until 2020 and the use of ARNIs was greatly impacted by the inexperience of clinicians and the financial situation of the patients. With the continuous improvement in clinicians' awareness and prescription of these drugs, adherence to performance measures has greatly improved, and the prescription rate of ARNIs increased to 66% by 2020.^34^ As for MRA therapy for patients with HFrEF, our study showed 84.9% of patients were treated with MRA, showing a large treatment gap compared with 33% of patients treated with MRA in the Change the Management of Patients with Heart Failure registry. This may be due to the fact that the patients included in our study were all NYHA III to IV patients with acute HF, and the proportion of patients with eGFR > 30 mL/min/1.73 m^2^ and serum potassium < 5.0 mmol/L was relatively high.

The rates of adherence to performance measures were also related to the medical quality of the hospital itself, including the hospital's care level, volume, academic status, and surgical capacity, as summarized in Table 2, which is consistent with the results of prior studies from other countries.^35^, ^36^, ^37^ An analysis of the quality of care for other diseases in China, including acute myocardial infarction, cerebral ischemic stroke, chronic obstructive pulmonary disease, and bacterial pneumonia, revealed that most hospitals had low adherence to performance measures, and that hospitals exhibited large heterogeneity in the quality of care.^38^ Our results may guide hospital policies aimed at organizational changes to reduce gaps in the care of patients with HF.

Most importantly, we have provided new information about the relationship between performance measures and outcomes in patients with HF. Higher adherence to performance measures was related to lower 1‐year mortality, even after adjusting for related confounding factors. This was consistent with the Organized Program to Initiate Lifesaving Treatment in Hospitalized Patients With Heart Failure registry study conducted in 2002, which suggested that increased adherence to performance measures was associated with reduced mortality and readmission rates. Specifically, in that study, patients with HF admitted to hospitals that provided specific process‐of‐care improvements had lower mortality and readmission rates than those who were not (34.8% vs. 38.2%).^39^ Similarly, the Danish Heart Failure Registry (DHFR) demonstrated that meeting process performance measures recommended in clinical guidelines for HF care was associated with a significant reduction in 1‐year mortality among patients with HF.^40^ However, we observed no statistically significant difference between the readmission rate and the compliance with performance measures, which may be explained by the fact that readmission for HF has more to do with socioeconomic determinants of health and the severity of the patient's condition than with the quality of care provided.

In addition to the performance measures included in our study, the performance and quality measures recommended in the guidelines also include exercise training and patient self‐care education. A systematic review showed that patient education for HF significantly influenced the readmission rate.^41^ However, further analysis of patients with HFrEF in the DHFR study demonstrated that patient education was associated with lower hospital bed daily use but not with readmission risk, while exercise training was associated with both reduced 1‐year mortality and readmission.^42^ However, in our observational study, exercise training and patient education were not provided to the patients. Therefore, the lack of significance in the relationship between the readmission rate and performance measures may also be affected by the lack of implementation of these two measures. In the future, exercise training and education for patients with HF should be increased to reduce mortality and readmission and further improve the quality of life.

The GWTG‐HF is a national hospital‐based quality improvement program launched by the AHA in 2005 to improve the quality of care provided for HF, which accounts for the large gap in the quality of such care between China and the United States.^13^ The gap in performance measures for patients with HF among different hospitals has gradually narrowed, and the patient prognosis has greatly improved with the development of the GWTG‐HF, which is unrelated to the volume and academic status of hospitals.^24^, ^43^ Nearly 20 years of experience with this program and our results provide lessons that may benefit China. The Chinese government could launch a hospital‐based medical quality monitoring system, publicly report performance measures, and financially reward or penalize hospitals, as appropriate. This may reduce the gap in the quality of medical care, stimulate the improvement of such care, and improve the prognosis of patients with HF in China.

## 5 LIMITATIONS

This study had several limitations. First, approximately half of the patients did not undergo LVEF assessment, possibly resulting in a portion of patients with HFrEF not being removed from the study. All echocardiograms were obtained in local hospitals, which may in part be the cause of heterogeneity in the LVEF. Currently, an accurate assessment of compliance with the Chinese guidelines is not feasible. Second, although the performance measures recommended in the guidelines also include exercise training and patient education, regrettably, these two measures were not included in this study. Finally, our study had a short follow‐up period (1 year) and did not provide further evidence of the relationship between performance measures and long‐term prognosis.

## 6 CONCLUSION

In conclusion, the quality of care for HF in China varies greatly among hospitals. Meeting process performance measures that reflect the recommendations of the clinical guidelines for HF care was associated with significantly reduced mortality among patients with HF.

## AUTHOR CONTRIBUTIONS

*Study concept and design and administrative, technical, or material support*: All authors. *Acquisition of data*: Shiyue Zheng, Li Li, Xiaoyan Zhao, Xiaofang Wang, and Xin Du. *Analysis and interpretation of data*: Shiyue Zheng, Ling Li, Chao Jiang, Liu He, Yiwei Lai, Xin Du, and Changsheng Ma. *Drafting of the manuscript*: Shiyue Zheng. *Critical revision of the manuscript for important intellectual content*: Shiyue Zheng and Jianzeng Dong. *Statistical analysis*: Shiyue Zheng, Liu He, Yiwei Lai. *Obtained funding and study supervision*: Jianzeng Dong.

## CONFLICT OF INTEREST STATEMENT

The authors declare no conflict of interest.

## Data Availability

Data cannot be shared for ethical/privacy reasons. The data underlying this article cannot be shared publicly due to the privacy of individuals who participated in the study. The data will be shared on reasonable request to the corresponding author. Dr Jianzeng Dong had full access to all the data in the study and took responsibility for the integrity of the data and the data analysis accuracy.
